# Preventive Health Screening during the COVID-19 Pandemic: A Cross-Sectional Survey among 102,928 Internet Users in Poland

**DOI:** 10.3390/jcm11123423

**Published:** 2022-06-14

**Authors:** Paulina Mularczyk-Tomczewska, Adam Żarnowski, Mariusz Gujski, Janusz Sytnik-Czetwertyński, Igor Pańkowski, Rafał Smoliński, Mateusz Jankowski

**Affiliations:** 1 Department of Public Health, Medical University of Warsaw, 02-097 Warsaw, Poland; paulina.mularczyk-tomczewska@wum.edu.pl (P.M.-T.); a.s.zarnowski@wp.pl (A.Ż.); 2 School of Public Health, Centre of Postgraduate Medical Education, 01-826 Warsaw, Poland; janusz.sytnik-czetwertynski@cmkp.edu.pl (J.S.-C.); mjankowski@cmkp.edu.pl (M.J.); 3 Emergency Department, Central Clinical Hospital of the Ministry of the Interior and Administration in Warsaw, 02-004 Warsaw, Poland; igor.pankowski@cskmswia.gov.pl; 4 Niepubliczny Zespół Zakładów Opieki Zdrowotnej Wigor, 05-120 Legionowo, Poland; rafal.smolinski@wigor.org

**Keywords:** screening, preventive medicine, laboratory testing, preventive care practices, COVID-19 pandemic, Poland

## Abstract

Preventive screening is a highly cost-effective public health intervention. The COVID-19 pandemic may impact preventive healthcare services. This study aimed to assess the frequency of preventive health screening, as well as to identify the factors associated with compliance with health screening guidelines among adults in Poland during the COVID-19 pandemic. This cross-sectional survey was carried out between October and December 2021, among Internet users in Poland. Respondents were asked about the last date that they performed seven different screening tests. Completed questionnaires were obtained from 102,928 adults aged 18–99 years, and 57.2% were female. The most common screening tests performed in the past 12 months were blood pressure measurement (83%), blood count (66.2%), and blood sugar (63.3%). Moreover, more than half of respondents had a urinalysis (53.1%) and lipid panel (55.1%) in the past 12 months. Out of 58,904 females, 69.2% had a cervical cytology in the past 3 years. Older age, having higher education, living in urban areas, being occupationally active, having at least one chronic disease, and visiting a doctor in the past 12 months were significantly associated (*p* < 0.001) with a higher level of compliance with screening guidelines. This study revealed a significant gap in the performance of preventive health screening.

## 1. Introduction

Preventive care is defined as the personal and community-wide efforts to prevent health problems before they occur [1,2]. Prevention can be divided into primary, secondary, and tertiary [3]. Primary prevention refers to the modification of risk factors associated with the development of the disease. Secondary prevention refers to the control of disease progression that has already occurred, and tertiary prevention mitigates the consequences of advanced disease for the functional state and quality of life [3].

Performing screening tests is an example of secondary prevention [3]. Early detection of diseases is crucial for inducing treatment at the early stage of the disease, which leads to improved survival and quality of life [4]. Moreover, preventive screening can significantly reduce treatment costs and the burden of the disease [5]. Hence, preventive healthcare services led to the improvement of the health status of the population, as well as the reduction of healthcare costs [6].

The most common screening test are laboratory tests [7,8]. A blood count is a basic laboratory blood test that allows for the detection of multiple diseases, as well as to monitor the health status before and after treatment [9]. A blood sugar (blood glucose) test is a common test that allows for the detection of pre-diabetes or diabetes [10]. A lipid panel is a simple blood test that is commonly used to assess the risk for cardiovascular diseases [11]. Screening laboratory tests also include urinalysis, which involves checking the appearance, concentration, and content of urine [12]. Urinalysis is commonly used to detect and manage kidney diseases, as well as some metabolic diseases or infections [12]. Except for the basic blood and urine test, another important screening test is cervical cytology [13,14]. Cervical cytology is well-established screening test to detect pre-invasive cervical lesions or cervical cancer [14]. Since cardiovascular diseases are a leading cause of death worldwide [15], preventive health care services also include non-laboratory screening tests, such as blood pressure measurement or an electrocardiogram [16]. Blood pressure measurement is a simple screening test that can be self-performed by the patient at home or a healthcare professional during a visit to the healthcare facility [17]. It is estimated that even 30% of adults worldwide have hypertension [18], so regular monitoring of blood pressure is crucial to detect hypertension at the early stage. An electrocardiogram is mostly used as a screening test among those who are at higher risk of cardiovascular diseases [19]. 

Screening recommendations differ across countries [20,21,22]. However, it is believed that basic screening tests, such as blood count, blood sugar tests, urinalysis, and blood pressure measurement should be performed annually [22,23]. The frequency of lipid panel tests, cervical cytology, and electrocardiograms depend on the individual risk factors; however, in most individuals, those screening tests should be carried out at least every 3 years [20,21,22,23]. 

Access to screening tests depends on the organization of the health care system in each country [24,25]. The World Health Organization recommends mass population screening for cervical, breast, and colorectal cancer [26]. However, due to the high cost-effectiveness, numerous countries had implemented screening for cardiovascular diseases, lung diseases, and diabetes [27,28]. Moreover, screening tests may be carried out as a part of occupational health screening [29]. Previously published data showed that the frequency of preventive health screening depends on educational level, health status, and health literacy levels [30,31,32]. Moreover, geographic accessibility to health facilities or medical laboratories, as well as the cost of tests (funding sources), may also affect the willingness to participate in screening [33,34]. 

Poland is an example of a European Union (EU) country with an insurance-based healthcare system [35]. All legally employed persons (and their spouses or children) are covered by compulsory health insurance that guarantees access to publicly funded healthcare services, including preventive care [35]. All the adults in Poland are regularly encouraged by healthcare professionals and key opinion leaders to participate in health screening. Insured individuals had access to free-of-charge screening tests that are administered by the general practitioner. The list of free-of-charge screening tests is regularly updated and includes blood tests, such as blood sugar (blood glucose), lipid panel, and blood count, as well as urinalyses and electrocardiograms [24]. Moreover, employed or self-employed individuals are obligated to complete occupational health screening regularly. Except for the basic screening tests (e.g., blood and urine tests), there are numerous national prevention programs for the early detection of cancers, cardiovascular diseases, or lung diseases that offer free-of-charge screening tests [36,37,38]. Additionally, local governments are offering screening services as a part of local public health programs [39]. Moreover, there is common access to private medical laboratories that offer self-paid screening tests (e.g., within a dedicated screening program targeted for particular risk groups, where the recommended tests are listed).

Despite the widespread access to basic screening tests in Poland, the frequency of testing is relatively low [36]. For example, it is estimated that approximately 25–30% of the eligible population took part in the cervical cancer screening program [36]. Moreover, the COVID-19 pandemic may decrease preventive healthcare utilization [40]. During the COVID-19 pandemic in Poland, there was limited access to healthcare, as well as a change in health priorities, which may have had a negative impact on the frequency of preventive health screening. Moreover, during the COVID-19 pandemic, public debate was dominated by the health topics that may shape pro-healthy behaviors. Detailed assessment of preventive care uptake is crucial for estimating the potential populational health effects of the COVID-19 pandemic (e.g., underdiagnosis of chronic diseases). However, there is a lack of scientific evidence regarding the impact of the COVID-19 pandemic on public attitudes towards preventive healthcare services and voluntary preventive screening. 

Therefore, the aim of this study was to assess the frequency of preventive health screening, as well as to identify the factors associated with compliance with health screening guidelines among adults in Poland during the COVID-19 pandemic.

## 2. Materials and Methods

### 2.1. Study Design and Population

This cross-sectional survey was carried out between 13 October and 27 December 2021, among Internet users in Poland. This study was carried out as a part of the project “Think about yourself—we check the health of Poles in a pandemic”, which was carried out by the Medical University of Warsaw, Wirtualna Polska Media SA (one of the largest web portals in Poland with approximately 13 million individual users every month), and HomeDoctor Sp. Z o.o. (one of the leading telemedicine companies in Poland) [41]. 

The computer-assisted web interview (CAWI) technique was used. The questionnaire was distributed through websites belonging to one of the leading Polish-language news and lifestyle Internet media house (Wirtualna Polska Media) [41]. The study questionnaire was available on the dedicated project website. Before starting the study, each respondent was informed of the objectives of the study and its course. The link to the questionnaire was placed on advertising/information banners on the websites (home page, as well as dedicated news, lifestyle, and health subpages). Participation in the study lasted 10–15 min.

### 2.2. Measures

The research tool was a questionnaire developed for the purpose of this study. The preparation of the questionnaire was preceded by an expert debate, which allowed for the identification of the most important health problems during the COVID-19 pandemic. The questionnaire included 32 questions concerning lifestyle, health choices, health condition, health problems, preventive health screening, and using health care during the COVID-19 pandemic. Moreover, questions about sociodemographic characteristics were addressed. 

Preventive health screening: Respondents were asked about preventive screening and laboratory testing, using the question: “Please indicate when you last performed the following screening tests: (1) blood pressure measurement; (2) blood sugar (blood glucose) test; (3) lipid panel; (4) blood count; (5) urinalysis; (6) electrocardiogram; (7) cervical cytology (question addressed only to women)”, with the following answer choices: “during the past 12 months”; “more than 1 year but not more than 2 years ago”; “more than 2 years ago but not more than 3 years ago”; “more than 3 years ago”; and “never”. 

Respondents who performed blood pressure measurement, blood sugar testing, blood count, and urinalysis in the past 12 months were qualified as those who perform screening tests, in accordance with national screening guidelines developed by the public health authorities [20,21,22]. Moreover, females who performed cervical cytology within last 3 years were classified as those who follow screening recommendations [42].

### 2.3. Ethics

Participation in the study was voluntary and anonymous. Informed consent was collected from all the participants. The study protocol was reviewed and approved by the Ethical Review Board at the Medical University of Warsaw, Warsaw, Poland (decision number AKBE/149/2021).

### 2.4. Statistical Analysis

The data were analyzed with SPSS V.28 (IBM Corp., Armonk, NY, USA). The distribution of categorical variables was shown by the frequencies and proportions. Statistical testing to compare categorical variables was completed using the independent sample chi-squared test. Associations between personal characteristics (gender, age, educational level, place of residence, occupational status (currently employed or self-employed were defined as active), presence of chronic diseases, visiting doctor in the past 12 months, and preventive health screening) and preventive health screening ((1) performing blood pressure measurement, (2) blood sugar test, (3) blood count, (4) urinalysis in the past 12 months, and (5) cervical cytology test in the past 36 months) were analyzed using multivariable logistic regression models. The strength of association was measured by the odds ratio (OR) and 95% confidence intervals (CIs). The level of statistical significance was set at 0.05.

## 3. Results

### 3.1. Characteristics of the Study Population

The completed questionnaires were obtained from 102,928 adults aged 18–99 years, and 57.2% were female. Half of the participants (49.5%) had a higher education, and 75.2% were occupationally active. Among the respondents, 42.4% had at least one chronic disease, and 70.4% had visited doctor during the past 12 months. The detailed characteristics of the study population are presented in Table 1. 

### 3.2. Preventive Health Screening among Adults in Poland

Most of the participants (83%) had their blood pressure measured in the past 12 months (Table 2). Two-thirds of respondents had a blood count test in the past 12 months, and 63.3% had a blood sugar test in the past 12 months. Moreover, more than half of respondents had urinalyses (53.1%) and lipid panels (55.1%) in the past 12 months. Among the respondents, 37.2% had an electrocardiogram in the past 12 months. Out of 58,904 females, 69.2% had cervical cytology in the past 3 years (Table 2).

There were statistically significant differences in the frequency of preventive health screening by sociodemographic factors (Table 3). Females, older respondents, those with higher education, inhabitants of large cities, and occupationally active individuals more often declared annual preventive health screening (Table 3). Moreover, individuals with at least one chronic disease, as well as those who had visited doctor in the past 12 months, more often declared that they performed screening tests in the past 12 months. The percentage of females in Poland who performed cervical cytology in the last 3 years also differed by sociodemographic factors (Table 4).

### 3.3. Factors Associated with Preventive Health Screening

The results of the multivariate logistic regression analyses are presented in Table 5 and Table 6. Females, compared to males, had higher odds of performing blood count tests (OR: 1.18; 95% CI: 1.14–1.21; *p* < 0.001) but lower odds of performing blood pressure measurements (OR: 0.91; 95% CI: 0.88–0.94; *p* < 0.001) or blood sugar test (OR: 0.93; 95% CI: 0.91–0.96; *p* < 0.001). Respondents aged 35 years and older had higher odds of preventive health screening, compared to those aged 18–34 years (*p* < 0.001). Respondents who lived in cities had higher odds of performing blood sugar tests, blood count, and urinalysis, compared to those living in rural areas (*p* < 0.001). Having higher education, being occupationally active, having at least one chronic disease, and visiting a doctor in the past 12 months were significantly associated with higher odds of preventive health screening (*p* < 0.001). Details are presented in Table 5.

Females under 65 years of age had higher odds of performing cervical cytology tests in the past 3 years (*p* < 0.001). Those who lived in cities had higher odds of performing cervical cytology tests, compared to those who lived in rural areas (*p* < 0.001). Having higher education, being occupationally active, having at least one chronic disease, and visiting a doctor in the past 3 years were significantly associated (*p* < 0.001) with higher odds of performing cervical cytology tests in the past 3 years. Details are presented in Table 6.

## 4. Discussion

To the authors’ best knowledge, this is the most up-to-date study on preventive health screening among more than 100,000 adults in Poland during the COVID-19 pandemic. This study showed significant gaps in compliance with health screening guidelines in Poland. Blood pressure measurement was the most common screening test declared by the respondents. However, two-thirds of adults in Poland declared preventive laboratory testing, such as blood count or blood sugar tests, in the past 12 months. Slightly more than half of the respondents had lipid panel tests or urinalyses in the past 12 months. Moreover, every tenth woman has never had cervical cytology. We observed a significant difference in the frequency of screening testing by sociodemographic factors. Older age, having higher education, living in urban areas, being occupationally active, having at least one chronic disease, and visiting a doctor in the past 12 months were significantly associated with a higher level of compliance with health screening guidelines.

The COVID-19 pandemic has led to reductions in access to healthcare [43]. As of March 2020, most of the healthcare facilities in Poland were actively involved in an anti-epidemic response [44]. Numerous hospitals were transformed into dedicated COVID-19 centers [45] and outpatient clinics; additionally, general practitioners offered teleconsultations, rather than in-person visits [46]. The lack of direct contact with healthcare professionals may lead to a decrease in the performance of preventive screening. In this study, 30% of respondents had not visited a doctor during the COVID-19 pandemic. Moreover, a significant percentage of respondents (30–45%) had not had basic screening tests during the COVID-19 pandemic. Findings from this study confirmed that the COVID-19 pandemic led to reductions in access to preventive healthcare services. 

Public attitudes towards participation in health screening depend on educational level, health status, health literacy levels, place of residence/geographic accessibility, and economic issues [30,31,32,33,34]. In this study, having higher education and living in urban areas were significantly associated with a higher level of compliance with health screening guidelines (performing screening tests annually), which is in line with previously published data. Additionally, currently employed or self-employed individuals were more likely to perform screening tests. We can hypothesize that the legal obligation to participate in occupational health screening may lead to the higher compliance with the screening guidelines observed in this group. Moreover, there were numerous workplace health promotion programs carried out by both public and private companies, which may have encouraged employees to participate in screening [47,48]. 

Health status is a significant factor that affects public attitudes towards participation in screening programs [31,32]. In this study, older age, having at least one chronic disease, and visiting a doctor in the past 12 months were also significantly associated with a higher level of compliance with health screening guidelines. A deterioration of health status may lead to changes in health behavior and an increase in the frequency of screening tests. Nevertheless, effective preventive care requires the regular performance of screening tests, regardless of the state of health.

Moreover, we can hypothesize that the significant differences in public attitudes towards participation in screening programs by age, health status, and occupational status may point to differences in primary and secondary screening uptake in Poland. Older individuals with chronic diseases who are occupationally passive are engaged in secondary prevention (i.e., preventing or delaying complications), contrary to younger, healthy individuals who are more focused on primary prevention. This finding underlines the need to implement a dedicated approach for encouraging different social groups to participate in screening tests. Self-perception of health risks may significantly influence the public’s behavior towards preventive health care services uptake.

Findings from this study showed that every tenth woman in Poland has never had cervical cytology. In Poland, cervical cytology (every 3 years) is funded by the public payer (free of charge) for all females aged 25–59 years [49]. Moreover, multiple educational campaigns offer free access to cervical cytology in local communities [50]. This study showed that the percentage of females who performed cytology within the last 3 years decreased with the age. Moreover, the frequency of cervical cancer screening increased with the size of the place of residence. There is an urgent need for educational campaigns on cervical cancer and screening targeting older females from rural areas and small cities without higher education. Moreover, local government, public opinion leaders, and healthcare professionals should be actively involved in screening programs [50].

This study has numerous practical implications for public health in Poland. First, our findings revealed a low level of compliance with health screening guidelines, especially during the COVID-19 pandemic, which may worsen the health status of the population and delay or omit the diagnosis of diseases, which has a negative impact on both the individual and populational levels. Second, significant differences in the performance of screening tests points underline the health inequalities. Basic screening tests allow for the early detection of diseases, which directly translates into the patient’s quality of life. The implementation of treatment at an early stage of the disease often allows for curing or reducing the negative effects of the disease. Screening is particularly important in the case of diabetes, which may lead to serious complications that significantly affect the quality of life and generate markable costs for healthcare. Education on health screening, as well as dedicated screening campaigns, should particularly be addressed to those respondents with a lower level of compliance with health screening guidelines. Third, public health actions are needed, in order to provide universal access to health screening, which is free of charge for vulnerable groups. Moreover, local governments should be involved in screening programs (especially in rural areas and small cities) to increase the percentage of individuals who perform screening tests regularly.

This study had several limitations. In this study, a random sample was used. Nevertheless, more than 100 thousand respondents completed the questionnaire, so the study population was one of the biggest reported in Poland. Second, preventive health screening was self-declared. Due to the lack of access to the medical records of the respondents, the performance of the screening test was not verified. Third, this study was limited to seven different screening tests, so further research is needed, in order to analyze public attitudes towards other screening tests (e.g., colonoscopy or breast cancer screening). However, the screening tests included in this study are basic tests that are widely available in Poland and can be performed, both under health insurance and self-paid, in private laboratories.

## 5. Conclusions

This study revealed a significant gap in the performance of preventive health screen-ing among adults in Poland. During the COVID-19 pandemic, every third Pole did not perform blood count or blood sugar tests, and almost half did not perform a urinalysis. The low level of compliance with health screening guidelines observed in this study may worsen the health status of the Polish population and exacerbate the health debt caused by the pandemic. Moreover, the significant difference in the frequency of screening testing by sociodemographic factors presented in this study underlines the need to provide personalized educational campaigns and promotion regarding preventive medicine and screening guidelines, which should be tailored to different social groups.

## Figures and Tables

**Table 1:** Characteristics of the study population (*n* = 102,928).

Variable	*n*	%
Gender		
Female	58,904	57.2
Male	44,024	42.8
Age (years)		
18–34	19,083	18.5
35–49	38,871	37.8
50–64	28,942	28.1
65+	16,032	15.6
Educational level		
Primary	2356	2.3
Vocational	10,051	9.8
Secondary	39,603	38.5
Higher	50,918	49.5
Place of residence		
Rural	21,353	20.7
City up to 50,000 residents	22,306	21.7
City from 51,000 to 100,000 residents	12,807	12.4
City from 101,000 to 200,000 residents	10,356	10.1
City from 201,000 to 500,000 residents	12,773	12.4
City above 500,000 residents	23,333	22.7
Occupational status		
Active	77,429	75.2
Passive	25,499	24.8
Presence of chronic diseases		
Yes	43,608	42.4
No	59,320	57.6
Visiting a doctor in the past 12 months		
Yes	72,471	70.4
No	30,457	29.6

**Table 2:** The frequency of preventive health screening among adults in Poland (*n* = 102,928).

Please Indicate When You Last Performed the Following Screening Tests	In the Past 12 Months	More than 1 Year but Not More Than 2 Years Ago	More than 2 Years Ago but Not More than 3 Years Ago	More than 3 Years Ago	Never
	% (95% CI)				
Blood pressure measurement	83.0 (82.8–83.3)	7.2 (7.1–7.4)	3.1 (3.0–3.2)	3.7 (3.6–3.8)	2.9 (2.8–3.0)
Blood sugar test	63.3 (63.0–63.6)	12.4 (12.3–12.7)	6.7 (6.6–6.9)	9.4 (9.2–9.5)	8.2 (8.0–8.3)
Lipid panel	55.1 (54.8–55.4)	13.2 (13.0–13.4)	7.3 (7.1–7.5)	9.2 (9.0–9.4)	15.2 (15.0–15.5)
Blood count	66.2 (65.9–66.5)	13.6 (13.3–13.8)	7.4 (7.2–7.5)	9.6 (9.5–9.8)	3.2 (3.1–3.4)
Urinalysis	53.1 (52.8–53.4)	16.8 (16.5–17.0)	9.9 (9.7–10.1)	16.0 (15.8–16.3)	4.2 (4.1–4.4)
Electrocardiogram	37.2 (36.9–37.5)	15.6 (15.3–15.8)	11.5 (11.3–11.7)	22.6 (22.3–22.8)	13.1 (13.0–13.4)
Cervical cytology (question addressed only to women; *n* = 58,904)	39.5 (39.1–39.9)	17.6 (17.3–18.0)	12.1 (11.8–12.4)	21.5 (21.2–21.9)	9.3 (9.0–9.5)

**Table 3:** Annual preventive health screening among adults in Poland by sociodemographic factors (*n* = 102,928).

Variable	Preventive Health Screening in the Past 12 Months—Percentage of Respondents Who Answered “Yes” by Sociodemographic Factors							
	Blood Pressure Measurement		Blood Sugar Test		Blood Count		Urinalysis	
	*n* (%)	*p*	*n* (%)	*p*	*n* (%)	*p*	*n* (%)	*p*
Gender								
Female	49,302 (83.7)	<0.001	37,839 (64.2)	<0.001	40,916 (69.5)	<0.001	32,243 (54.7)	<0.001
Male	36,156 (82.1)		27,329 (62.1)		27,219 (61.8)		22,377 (50.8)	
Age (years)								
18–34	13,866 (72.7)	<0.001	9604 (50.3)	<0.001	10,654 (55.8)	<0.001	7665 (40.2)	<0.001
35–49	30,990 (79.7)		22,442 (57.7)		24,107 (62.0)		18,636 (47.9)	
50–64	25,780 (89.1)		20,455 (70.7)		20,717 (71.6)		17,265 (59.7)	
65+	14,822 (92.5)		12,667 (79.0)		12,657 (78.9)		11,054 (68.9)	
Educational level								
Primary	1507 (64.0)	<0.001	1093 (46.4)	<0.001	1139 (48.3)	<0.001	922 (39.1)	<0.001
Vocational	7712 (76.7)		5749 (57.2)		5956 (59.3)		4837 (48.1)	
Secondary	32,745 (82.7)		25,079 (63.3)		25,684 (64.9)		20,763 (52.4)	
Higher	43,494 (85.4)		33,247 (65.3)		35,356 (69.4)		28,098 (55.2)	
Place of residence								
Rural	17,409 (81.5)	<0.001	12,754 (59.7)	<0.001	13,283 (62.2)	<0.001	10,390 (48.7)	<0.001
City up to 50,000 residents	18,858 (84.5)		14,361 (64.4)		14,755 (66.1)		11,808 (52.9)	
City from 51,000 to 100,000 residents	10,679 (83.4)		8255 (64.5)		8650 (67.5)		6884 (53.8)	
City from 101,000 to 200,000 residents	8569 (82.7)		6593 (63.7)		6853 (66.2)		5547 (53.6)	
City from 201,000 to 500,000 residents	10,586 (82.9)		8118 (63.6)		8563 (67.0)		6923 (54.2)	
City above 500,000 residents	19,357 (83.0)		15,087 (64.7)		16,031 (68.7)		13,068 (56.0)	
Occupational status								
Active	63,424 (81.9)	<0.001	47,436 (61.3)	<0.001	49,987 (64.6)	<0.001	39,332 (50.8)	<0.001
Passive	22,034 (86.4)		17,732 (69.5)		18,148 (71.2)		15,288 (60.0)	
Presence of chronic diseases								
Yes	39,280 (90.1)	<0.001	32,244 (73.9)	<0.001	33,862 (77.7)	<0.001	27,529 (63.1)	<0.001
No	46,178 (77.8)		32,924 (55.5)		34,273 (57.8)		27,091 (45.7)	
Visiting a doctor in the past 12 months								
Yes	63,130 (87.1)	<0.001	50,177 (69.2)	<0.001	53,915 (74.4)	<0.001	43,639 (60.2)	<0.001
No	22,328 (73.3)		14,991 (49.2)		14,220 (46.7)		10,981 (36.1)	

**Table 4:** The percentage of females in Poland who performed cervical cytology in the last 3 years by sociodemographic factors (*n* = 58,904).

Cervical Cytology Test in the Last 3 Years—Percentage of Respondents Who Answered “Yes” by Sociodemographic Factors (*n* = 58,904)		
Variable	*n* (%)	*p*
Age (years)		
18–34	7003 (66.4)	<0.001
35–49	16,544 (75.9)	
50–64	12,315 (69.5)	
65+	4908 (55.6)	
Educational level		
Primary	657 (52.2)	<0.001
Vocational	2780 (61.7)	
Secondary	14,263 (64.7)	
Higher	23,070 (74.2)	
Place of residence		
Rural	8372 (67.2)	<0.001
City up to 50,000 residents	8014 (67.3)	
City from 51,000 to 100,000 residents	5175 (69.9)	
City from 101,000 to 200,000 residents	4191 (70.1)	
City from 201,000 to 500,000 residents	5196 (69.8)	
City above 500,000 residents	9822 (71.6)	
Occupational status		
Active	30,213 (72.8)	<0.001
Passive	10,557 (60.6)	
Presence of chronic diseases		
Yes	19,651 (70.3)	<0.001
No	21,119 (68.2)	
Visiting a doctor in the past 12 months		
Yes	32,331 (73.4)	<0.001
No	8439 (56.9)	

**Table 5:** Factors associated with preventive health screening in the past 12 months among adults in Poland (*n* = 102,928)—multivariable logistic regression model.

Variable	Factors Associated with Preventive Health Screening in the Past 12 Months among Adults in Poland							
	Blood Pressure Measurement		Blood Sugar Test		Blood Count		Urinalysis	
	*p*-Value	OR (95% CI)	*p*-Value	OR (95% CI)	*p*-Value	OR (95% CI)	*p*-Value	OR (95% CI)
Gender								
Female	<0.001	0.91 (0.88–0.94)	<0.001	0.93 (0.91–0.96)	<0.001	1.18 (1.14–1.21)	0.97	1.00 (0.97–1.03)
Male	Reference	Reference	Reference	Reference	Reference	Reference	Reference	Reference
Age (years)								
18–34	Reference	Reference	Reference	Reference	Reference	Reference	Reference	Reference
35–49	<0.001	1.30 (1.25–1.36)	<0.001	1.21 (1.17–1.25)	<0.001	1.12 (1.08–1.16)	<0.001	1.24 (1.20–1.29)
50–64	<0.001	2.80 (2.66–2.94)	<0.001	2.17 (2.08–2.25)	<0.001	1.79 (1.72–1.86)	<0.001	2.05 (1.98–2.14)
65+	<0.001	4.11 (3.81–4.42)	<0.001	3.29 (3.11–3.47)	<0.001	2.64 (2.50–2.79)	<0.001	2.94 (2.79–3.10)
Having higher education								
Yes	<0.001	1.48 (1.43–1.53)	<0.001	1.23 (1.20–1.26)	<0.001	1.33 (1.29–1.37)	<0.01	1.21 (1.17–1.24)
No	Reference	Reference	Reference	Reference	Reference	Reference	Reference	Reference
Place of residence								
Rural	Reference	Reference	Reference	Reference	Reference	Reference	Reference	Reference
City up to 50,000 residents	<0.05	1.07 (1.02–1.13)	<0.001	1.09 (1.05–1.14)	<0.001	1.10 (1.05–1.15)	<0.001	1.08 (1.04–1.12)
City from 51,000 to 100,000 residents	0.3	1.03 (0.97–1.10)	<0.001	1.15 (1.10–1.21)	<0.001	1.22 (1.16–1.28)	<0.001	1.17 (1.12–1.22)
City from 101,000 to 200,000 residents	0.3	0.97 (0.91–1.03)	<0.001	1.09 (1.04–1.15)	<0.001	1.11 (1.06–1.17)	<0.001	1.14 (1.08–1.20)
City from 201,000 to 500,000 residents	0.5	0.98 (0.92–1.04)	<0.001	1.08 (1.03–1.13)	<0.001	1.16 (1.10–1.21)	<0.001	1.17 (1.12–1.23)
City above 500,000 residents	<0.05	0.95 (0.90–0.99)	<0.001	1.11 (1.07–1.16)	<0.001	1.22 (1.17–1.27)	<0.001	1.24 (1.17–1.24)
Occupational status								
Active	<0.001	1.17 (1.12–1.23)	<0.001	1.13 (1.08–1.17)	<0.001	1.19 (1.14–1.23)	<0.001	1.06 (1.03–1.10)
Passive	Reference	Reference	Reference	Reference	Reference	Reference	Reference	Reference
Presence of chronic diseases								
Yes	<0.001	1.86 (1.79–1.93)	<0.001	1.71 (1.66–1.76)	<0.001	1.86 (1.80–1.91)	<0.001	1.49 (1.45–1.53)
No	Reference	Reference	Reference	Reference	Reference	Reference	Reference	Reference
Visiting a doctor in the past 12 months								
Yes	<0.001	2.23 (2.16–2.31)	<0.001	2.15 (2.09–2.21)	<0.001	2.99 (2.90–3.08)	<0.001	2.52 (2.45–2.59)
No	Reference	Reference	Reference	Reference	Reference	Reference	Reference	Reference

**Table 6:** Factors associated with cervical cytology testing in the past 3 years among adult females in Poland (*n* = 58,904)—multivariable logistic regression model.

Variable	Factors Associated with Performing Cervical Cytology in the Past 3 Years	
	*p*-Value	OR (95% CI)
Age (years)		
18–34	<0.001	1.42 (1.33–1.52)
35–49	<0.001	2.07 (1.95–2.21)
50–64	<0.001	1.68 (1.58–1.78)
65+	Reference	Reference
Having higher education		
Yes	<0.001	1.46 (1.40–1.51)
No	Reference	Reference
Place of residence		
Rural	Reference	Reference
City up to 50,000 residents	0.3	1.03 (0.97–1.09)
City from 51,000 to 100,000 residents	<0.001	1.15 (1.08–1.23)
City from 101,000 to 200,000 residents	<0.001	1.16 (1.09–1.25)
City from 201,000 to 500,000 residents	<0.001	1.15 (1.08–1.22)
City above 500,000 residents	<0.001	1.23 (1.16–1.30)
Occupational status		
Active	<0.001	1.32 (1.26–1.38)
Passive	Reference	Reference
Presence of chronic diseases		
Yes	<0.001	1.10 (1.06–1.15)
No	Reference	Reference
Visiting a doctor in the past 12 months		
Yes	<0.001	2.06 (1.98–2.14)
No	Reference	Reference

## Data Availability

Data are available on reasonable request. The data set used to conduct the analyses are available from corresponding author on reasonable request.
